# Relation between high density lipoprotein particles concentration and cardiovascular events: a meta-analysis

**DOI:** 10.1186/s12944-018-0732-6

**Published:** 2018-06-19

**Authors:** YuJing Wu, ZhiJuan Fan, YaQiong Tian, Shuang Liu, ShuYe Liu

**Affiliations:** Third Central Hospital of Tianjin, Tianjin Institute of Hepatobiliary Disease, Tianjin Key Laboratory of Artificial Cell, Artificial Cell Engineering Technology Research Center of Public Health Ministry, No. 83, Jintang Road, Hedong District, Tianjin, China

**Keywords:** HDL, Particle, Cardiovascular events, Meta analysis

## Abstract

**Background:**

Trails aimed at raising high density lipoprotein(HDL) cholesterol concentration failed to make better cardiovascular outcomes. HDL particles may be better biomarkers reflecting properties of HDL. This meta-analysis was conducted to evaluate the relation between blood HDL particles level and cardiovascular events.

**Methods:**

PubMed and other databases were searched for eligible studies and NewCastle-Ottawa Quality Assessment Scale(NOS) was used to assess the quality of included studies. A random or fixed-effect model was applied to calculate the pooled hazard ratio(HR).

**Results:**

Twelve studies were finally included. The pooled HR(95%confidence interval) for per standard deviation(SD) increment and top quartile versus bottom quartile were 0.79(0.72,0.86) and 0.65(0.57,0.75), respectively. Subgroup analysis suggested that HR was significantly lower in subjects with a cardiovascular disease(CVD) history than that of people without established CVD. Subclass analysis indicated that HRs for per SD increment of small(0.85) and medium(0.84) HDL particles were significantly lower than that of large HDL particles(0.96).

**Conclusions:**

HDL particle level in blood was inversely related to CVD events, indicating that HDL particles maybe a protective factor in patients with CVD, thus making HDL particles a potential biomarker and therapy target.

## Background

It is well acknowledged that high density lipoproteins (HDL) play a role in anti-atherosclerosis and protect against the development of cardiovascular diseases (CVD). Serum concentrations of HDL cholesterol (HDL-C), the most widely used biomarker about HDL, are inversely related to risk of atherosclerotic CVD. But trails aimed at increasing HDL-C failed to improve CVD outcomes [1, 2], raising the question whether HDL-C is the best biomarker for assessing the relationship between HDL and CVD risk.

Indeed, HDL is a kind of heterogeneous population of lipoproteins, representing a spectrum of lipoprotein particles ranging 1.063–1.21 g/ml in density and 7–12 nm in size. Since HDL-C may not fully represent the properties of HDL, alternative biomarkers and parameters of HDL, such as the concentration of HDL particles (HDL-P), HDL size and apolipoprotein A1 (apoA1), are getting more attentions. Several studies evaluated the association between HDL-P and CVD outcomes, indicating that HDL-P may be a parameter making more sense in predicting CVD outcomes than HDL-C. In this study, we performed a meta-analysis to explore the relationship between HDL-P and CVD outcomes.

## Methods

### Selection criteria

Clinical studies were considered eligible if they satisfied the following inclusion criteria: 1) the studies about the association between concentration of HDL-P in plasma or serum and CVD outcomes; 2) Hazard ratio(HR) or odds ratio(OR) adjusted for potential influencing factors with 95% confidence interval(CI) should be available; 3) more than 50 patients should be involved in the study. Studies were excluded on any of the following criteria: 1) review articles or case reports; 2) If studies were published by the same group with overlapping patient populations, the most recent one was selected.

### Search strategy

We searched the PubMed, Cochrane Library, China National Knowledge Infrastructure(CNKI) and Weipu(VIP) database using varying combinations of the following keywords: “high density lipoprotein”, “HDL” “subclasses”, “particles”, “subpopulation”, “subfraction”; “CVD”, “cardiovascular diseases”, “cardiovascular events”, “cardiac events”. The results were limited to English and Chinese language. The last search update was performed on November 20,2016. References of all eligible studies were manually searched for additional eligible studies.

### Quality assessment and data extraction

The quality of articles was assessed using NewCastle-Ottawa Quality Assessment Scale (NOS) [3, 4], by two investigators independently. Disagreement was resolved by discussion and consulting a third investigator.

Two investigators collected following information from all the included articles independently: first author’s name, publication year, study design, sample size, basic status of subjects(country, age, disease status, treatment), detection method for HDL-P, definition of outcome events, follow-up time, HR/OR for total HDL-P and HDL-P subclasses if applicable.

### Statistical methods

STATA 11.0 software was used to perform all statistical analyses. Forest plots were used to assess the pooled HR of HDL-P concentration for CVD events. The *I*^*2*^ test was performed to estimate the heterogeneity between articles. *I*^*2*^ more than 50% indicated significant heterogeneity and a random-effect model was applied. Otherwise, a fixed-effect model was used. Subgroup analysis was conducted to determine the source of heterogeneity. Heterogeneity was also investigated by sensitivity analysis, which was performed by removing each study sequentially to assess the impact of each study. Potential publication bias was assessed with funnel plots and the Begg’s test. *P* value < 0.05 was considered as statistically significance.

## Results

### Eligible studies and quality assessment

**Fig. 1:**
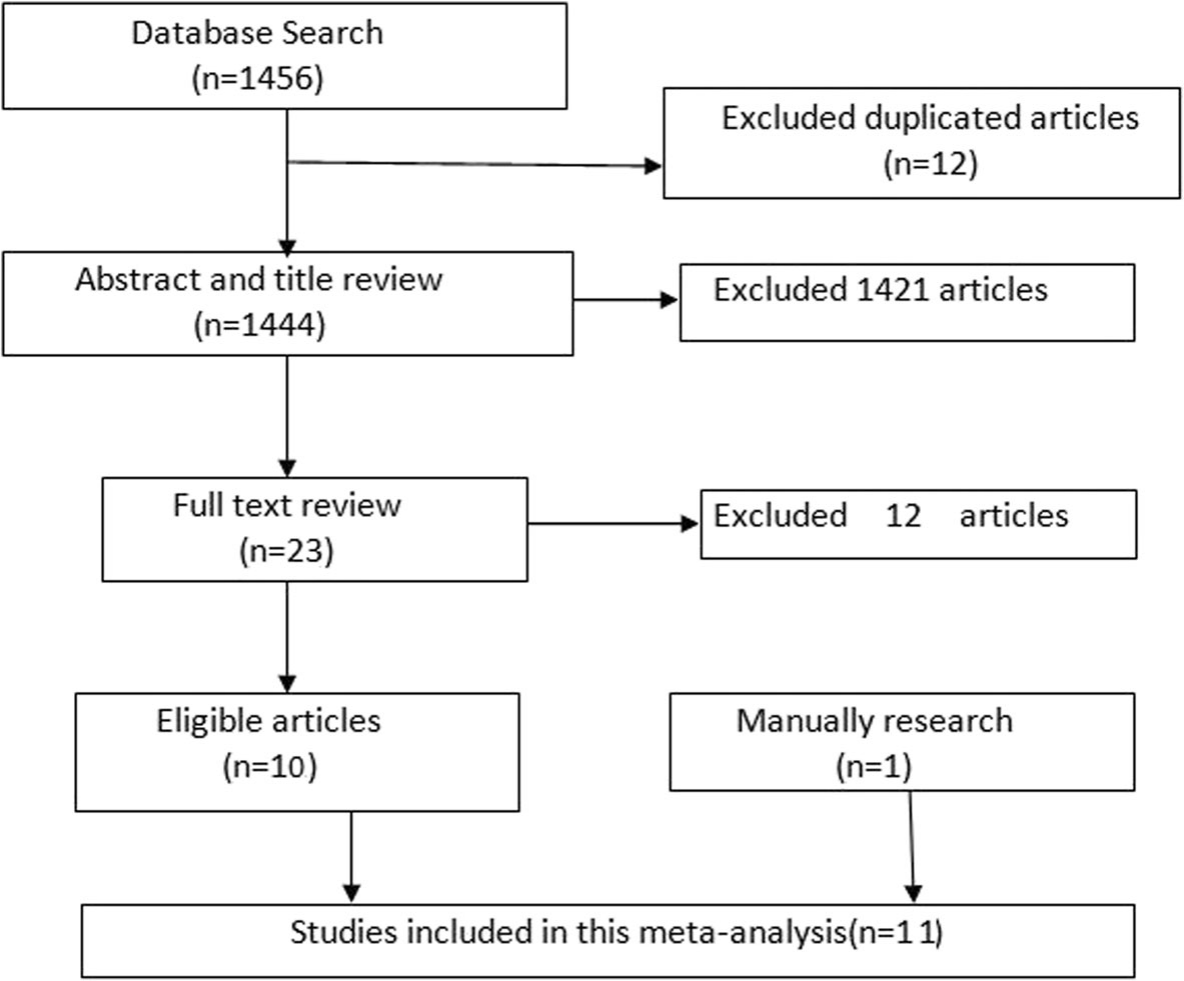
Flowchart of the included studies

Flowchart of study selection was illustrated in Fig. 1. Eleven studies [5–15] were finally included in this meta-analysis. The basic characteristics and quality assessment result of the eligible articles were shown in Table 1.

**Table 1:** Characteristics of the Eligible studies

First Author	Year of publication	Study design	Sample size(case+control/events+free of events)	Area	Age(years)	Quality score
Mackey	2012	cohort study	5597(227 + 5370)	US	mean of 61.5	9
Hsia	2008	nested case-control study	group1:404(202 + 202)	US	50 to 79	7
			group2:304(152 + 152)			
Akinkuolie	2014	cohort study	model 1: 25706(947 + 24,759); model 2: 25232(911 + 24,321)	US	Free of events: median of 52.6; Events:median of 57.9	9
Berger	2012	nested case-control study	1372(686 + 686)	US	median of 69	8
Duprez	2009	nested case-control study	728(248 + 480)	cross-country	median of 49	5
Kuller	2007	case control study	428(214 + 214)	US	mean of 51.3	8
Otvos	2006	nested case-control study	1061(364 + 697)	US	mean of 64.4	7
Chandra	2015	cohort study	1977	US	not given	8
Parish	2012	cohort study	20,021(1796 + 18,225)	UK	40 to 80	8
Musunuru	2009	cohort study	4594(337 + 4257)	Sweden	not given	9
Harchaoui	2009	nested case–control study	2223(822 + 1401)	UK	mean of 65	9

Since two groups in Hsia’s study [12] used different subjects without overlapping, we treated this study as two independent ones in following analysis. Two models were established in Akinkuolie’s [5] study because of different influencing factors adjusted for. There existed overlapping subjects. The whole sample size was 26,332(data not shown). Eleven articles with 63,064 subjects were involved in this meta-analysis. Seven out of the 11 studies were conducted in America.

We used NOS to evaluate the quality of eligible articles. The full score is nine in NOS system. Duprez’s study [9] scored as five, ranking normal in quality assessment. Others got scores higher than 6, ranking as high quality. And there were four studies getting full marks in NOS quality assessment.

### Correlation between total HDL-P concentration and CVD events

**Fig. 2:**
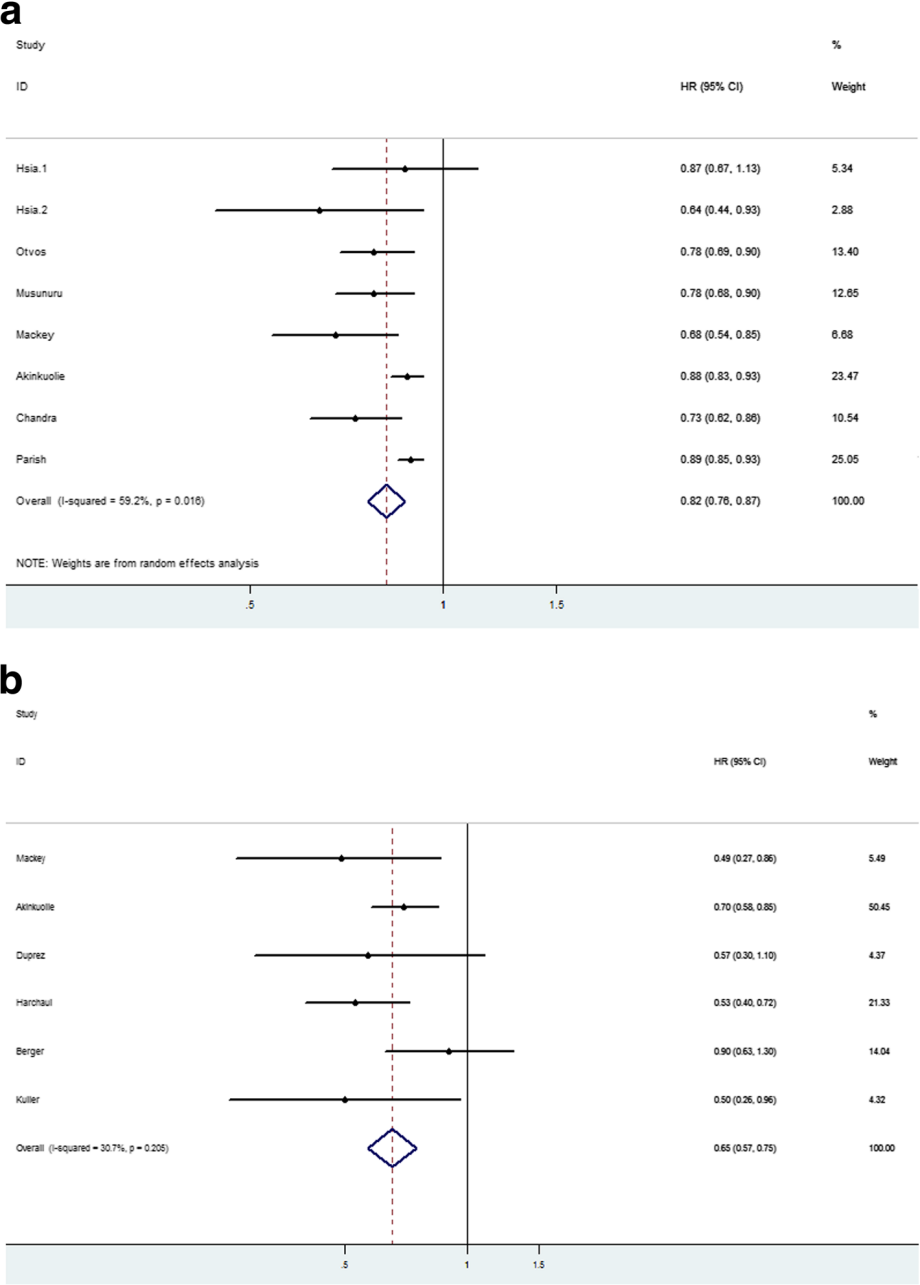
Forest plots of HR between total HDL-P concentration and CVD events. **a** shows the combined HR for per SD increment using a random-effect model for I^2^ = 59.2%, *P* = 0.004. **b** shows the pooled HR for Q4 vs Q1 by a fixed-effect model with I^2^ = 30.7%, *P* = 0.205

As shown in Table 2, HR or OR was calculated for per standard deviation(SD) increment or top quartile (Q4) versus bottom quartile (Q1) and we calculated pooled HR(OR) for both per SD increment and Q4 vs Q1. The combined HR for per SD increment was 0.82(95%CI:0.76,0.87) using a random-effect model for I^2^ = 59.2%, *P* = 0.004(Fig. 2a). The pooled HR for Q4 vs Q1 was 0.65 (95%CI: 0.57,0.75) calculated by a fixed-effect model with I^2^ = 30.7%, *P* = 0.205(Fig. 2b). Both indicated that a higher HDL-P level was reversely related with CVD events. The forest plots were drawn in Fig. 2.

**Table 2:** Correlation between total HDL-P concentration and CVD events

Author	Baseline of subjects	Adjusted for	Events	Follow-up time	HR(95%CI) or OR for 1-SD increment	HR(95%CI) or OR for Q4 vs Q1
Mackey	MESA articipants without self-reported CVD,pregnancy, cancer, cognitive impairment, or weight > 136 kg,lipid-lowering medication use,TG > 400 mg/dl	**model1**:age,sex,ethnicity,hypertension,smoking; **model2**: model1 + HDL-C,LDL-P,LDL-C,TG	myocardial infarction, CHD death, resuscitated cardiac arrest, or definite or probable angina (followed by revascularization)	mean of 6.0 years	model1:0.70(0.59–0.82); model2: 0.68(0.54–0.85)	model 1: 0.46(0.30–0.71);model2: 0.49(0.27–0.86)
Hsia(group1)	postmenopausal women with intact uterus in Estrogen Plus Progestin Trial	age, treatment arm, smoking, alcohol use, diabetes, hypertension	CHD MI/coronary death	4 years	0.87 (0.67–1.13)	–
Hsia(group2)	postmenopausal women with prior hysterectomy in Estrogen Alone Trial	age, treatment arm, smoking, alcohol use, diabetes, hypertension	CHD MI/coronary death	4 years	0.64 (0.44–0.93)	–
Akinkuolie	subjects in the WHS free of self-reported CVD or cancer or lipid-lowering medications	**model 1**:age, race,blood pressure, smoking, menopausal status,hormone replacement therapy,and treatment assignment.**model 2**: model 1 + BMI,diabetes,LDL-C, LDL-P,TG and other HDL subclasses.	nonfatal MI, percutaneous coronary,intervention, coronary artery bypass grafting, and CHD death	median of 17 years	model1: 0.91(0.86–0.97); model2: 0.88(0.83–0.93)	model 1:0.77(0.64–0.92); model2:0.70(0.58–0.85)
Berger	postmenopausal women from the WHI-OS with no prior history of MI or stroke	smoking status,BMI,systolic blood pressure,use of anti-hypertensive medication,diabetes and physical activity	Ischemic stroke	mean follow-up of 7.9 years	–	0.90(0.63–1.30)
Duprez	HIV-infected patients	**model1**:age, race, HIV-RNA and ART status, smoking, prior CVD, diabetes, use of BP-lowering drugs, use of lipid-lowering drugs, hepatitis co-infection, CD4+, BMI and major baseline ECG abnormalities;**model2**:model1 + LDL and triglycerides+D-dimer, IL-6 and hsCRP	non-fatal CHD events (defined as clinical and silent myocardial infarction, coronary revascularization and coronary artery disease requiring drug treatment), non-fatal atherosclerotic non-CHD (defined as stroke and peripheral arterial disease), congestive heart failure and fatal CVD (defines as CVD death and unwitnessed death)	–	–	model1: 0.41(0.2–0.7); model2: 0.57(0.3–1.1)
Kuller	men with metabolic syndrome within the MRFIT	white blood cell count,smoking status	CHD death	18 years	–	0.50(0.26–0.96)
Otvos	men with an established diagnosis of CHD in the VA-HIT	treatment,age, hypertension,smoking,BMI, diabetes	a nonfatal MI or CHD death	median of 5.1 years	0.78(0.69–0.90)	–
Chandra	participants from the Dallas Heart Study not taking any lipid lowering medication or hormone replacement therapy,free from malignancy, connective tissue disease, or HIV	**model1**:age, sex, ethnicity, hypertension, diabetes, smoking, BMI, non-HDL-C, logTG, any lipid-lowering therapy, hormone replacement therapy, menopause, alcohol intake, and history of CHD at baseline; **model2**:model1 + HDL-C	non-fatal myocardial infarction, stroke,coronary artery bypass graft (CABG), percutaneous coronary intervention, or cardiovascular death	median of 9.3 years	model1: 0.75(0.65–0.86); model2: 0.73(0.62–0.86)	–
Parish	high-risk individuals in the MRC/BHF HPS.A nonfasting blood total cholesterol concentration of at least 3.5 mmol/L (135 mg/dL) and either had a previous diagnosis of CHD, cerebrovascular disease, other occlusive disease of noncoronary arteries, or diabetes mellitus (type I or II) or men 65 years of age undergoing treatment for hypertension	**model1**:age, sex, simvastatin and vitamin allocation, smoking, prior disease, systolic blood pressure, estimated glomerular filtration rate, medication, and N-terminal pro-B-type natriuretic peptide;**model2** = model1 + LDL-P	nonfatal MI or coronary death other than death from heart failure or sudden death	mean of 5.3 years	model1: 0.88(0.83–0.92); model2: 0.89(0.85–0.93)	–
Musunuru	healthy people in the MDC-CC. Subjects with prior MI/stroke or on baseline lipid-lowering therapy were excluded	age, gender, systolic blood pressure, use of antihypertensivemedications,diabetes status, and current smoking status	myocardial infarction, stroke, and death from coronary heart disease, a secondary coronary endpoint of myocardial infarction and death from coronary heart disease	mean of 12.2 years	0.78(0.68–0.90)	–
Harchaoui	participants in EPIC-Norfolk cohort.All individuals who reported a history of heart attack or stroke or use of lipid-lowering drugs at the baseline clinic visit were excluded	**model1**:smoking,myeloperoxidase, paraoxonase 1, and C-reactive protein levels; **model2** = smoking+apol poprotein B and logTG	fatal or nonfatal CAD which was defined as codes 410 to 414 according to the International Classification of Diseases, Ninth Revision	average of 6 years	–	model1: 0.78(0.59–1.03); model2: 0.53(0.40–0.72)

*TG* triglyeride, *CHD* coronary heart disease, *NMR* nuclear magnetic resonance, *LDL-P* low density lipoprotein particles, *LDL-C* low density lipoprotein cholesterol, *MI* myocardial infarction, *BMI* body mass index, *CAD* coronary artery disease, *HIV* Human immunoddficiency virus, *ART* antiretroviral therapy, *ECG* electrocardiography, *hsCRP* high sensitivity C-Reactive Protein, *MESA* ulti-ethnic study of atherosclerosis, *WHS* omen’s Health Study, *WHI-OS* Women’s Health Initiative Observational Study, *MRFIT* Multiplle Risk Factor Intervention Trial, *VA-HIT* Veterans Affairs High-Density Lipoprotein Intervantion Trial, *HPS* Heart Protection Study, *MDC-CC* Malmö Diet and Cancer Study, *EPIC* European Prospective Investigation into Cancer and Nutrition

### Assessment of heterogeneity

Evidence of heterogeneity existed in HR for per SD increment. No heterogeneity was detected in HR for Q4 vs Q1. So subgroup meta-analysis was conducted to explore the source of heterogeneity in studies about HR for per SD increment. We divided studies into different subgroups according to study design, baseline status(patients with a history of CVD or not), influencing factors adjusted for(adjusting for lipids or not). There were two models in each of Mackey’s [7], Akinkuolie’s [5], Chandra’s [6] and Parish’s [13] studies. Compared to model 1, model 2 was additionally adjusted for lipids. Table 3 shows the meta-analysis results of subgroups. No grouping basis could explain heterogeneity fully, but case-control studies and studies only about females got I^2^ < 50% and *P* > 0.1, indicating insignificant heterogeneity.

**Table 3:** Meta-analysis results of subgroups

Subgroups		I^2^(%)	*P*	Pooled HR	95% CI
study design	case-control	0.0	0.418	0.78	0.70,0.88
	cohort	69.0	0.001	0.83	0.79,0.88
sex	female only	18.6	0.297	0.89	0.84,0.93
	both	73.3	0.001	0.80	0.74,0.86
baseline status	no CVD history	74.7	0.003	0.82	0.74,0.89
	partly with CVD	60.0	0.029	0.83	0.78,0.89
influencing factors adjusted for	adjusted for lipids	70.1	0.018	0.84	0.77,0.91
	not adjusted for lipids	65.2	0.005	0.81	0.76,0.87

Also, sensitivity analysis was conducted by omitting the studies one by one and repeating the meta-analysis. But heterogeneity remained significant with single study removal. Heterogeneity became insignificant with the removal of Akinkuolie’s [5] study together with Parish’s [13] (I^2^ = 0.0%, *P* = 0.658). The pooled HR was 0.76(95% CI: 0.71,0.82) with no significant difference from the HR with these two articles included.

### Meta-analysis about HDL-P subclasses

**Fig. 3:**
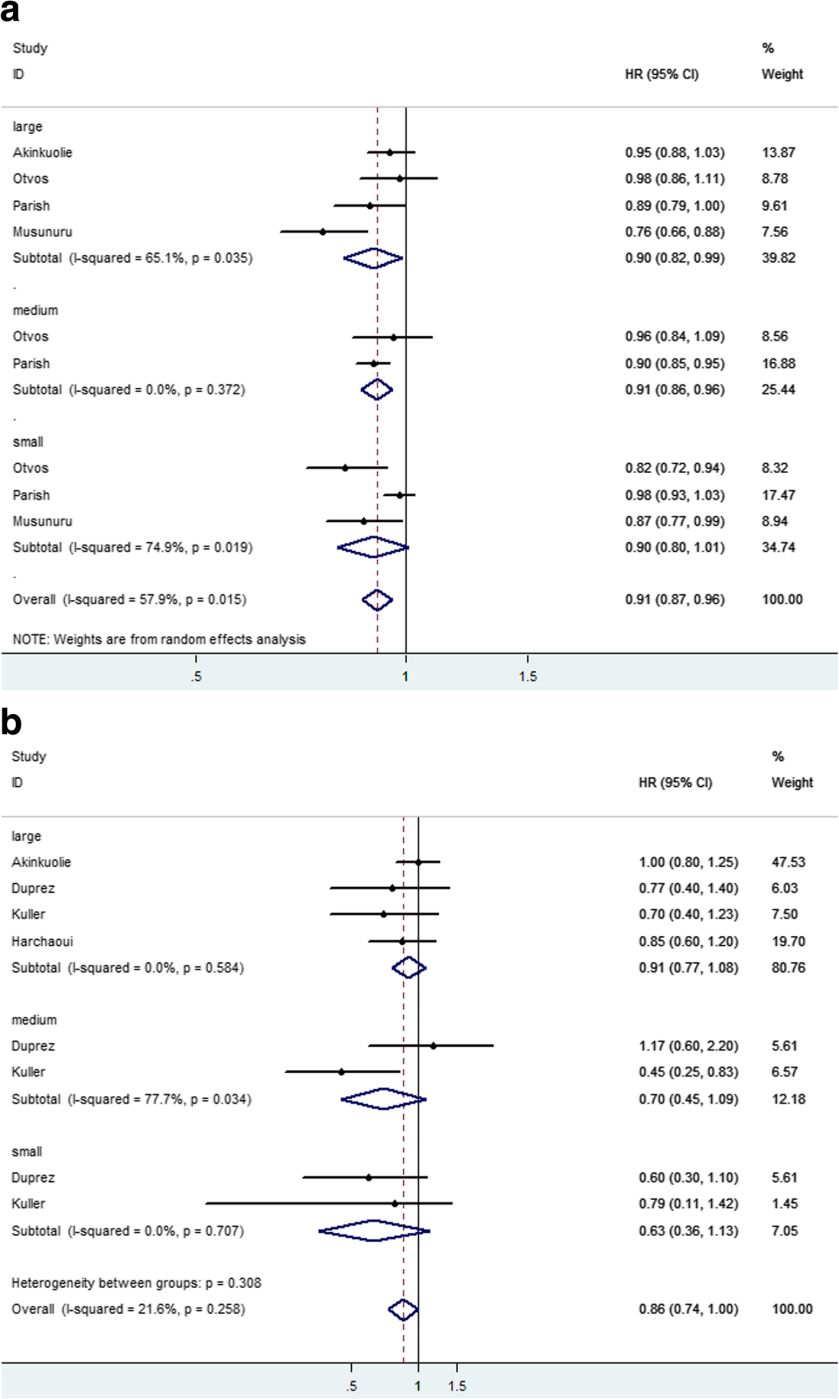
Forest plots for small, medium and large HDL-P. **a** HRs were calculated for per SD increment. **b** HRs were calculated for Q4 vs Q1

We also collected the data about HR or OR for HDL-P subclasses and CVD events. The diameter ranges of large, medium and small HDL-P were 8.8–14.0 nm, 8.2–8.8 nm, 7.3–8.2 nm, respectively. Seven articles analyzed the relation between levels of large HDL-P and CVD events. Four studied the relation between levels of medium HDL-P and CVD events. Five studied the relation between levels of small HDL-P with CVD events. HRs was calculated for per SD increment and Q4 versus Q1. Forest plots were shown in Fig. 3. In Fig. 3a, HRs were calculated for per SD increment. Subtotal HRs of large, medium and small HDL-P were 0.90(0.82,0.99), 0.91(0.86,0.96) and 0.90(0.80,1.01), respectively. In Fig. 3b, no significant difference was observed in HRs of HDL-P subclasses.

### Assessment of publication bias test

**Fig. 4:**
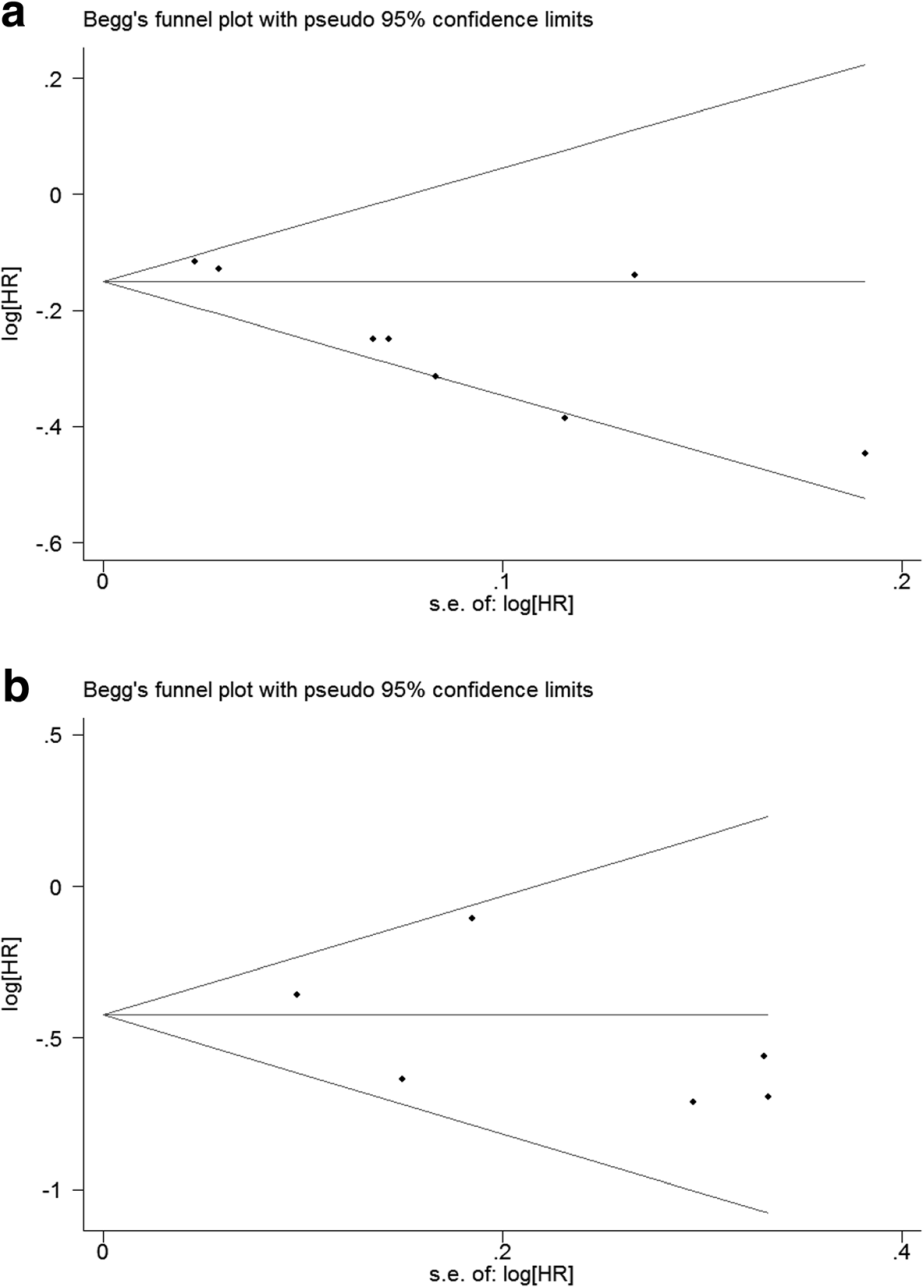
funnel plots of included studies. **a** shows the Begg's funnel plots of studies about HR calculated for per SD increment. **b** shows the Begg's funnel plots of studies about HR calculated for Q4 vs Q1

As Fig. 4 shows, studies included were symmetrical. As shown in Fig. 4,the Begg’s funnel plots of eight studies about HR calculated for per SD increment(Fig. 4a) and Q4 vs Q1(Fig. 4b) were almost symmetric(*P* = 0.108, 1.00, respectively),suggesting a low likelihood of publication bias.

## Discussion and conclusions

It is well established that serum HDL takes a cardioprotective role and is reversely related with CVD events risk. Functions of HDL include its role in reverse cholesterol transport (RCT), anti-oxidation and anti-inflammation. In this meta-analysis, we searched and analyzed studies about the value of blood HDL-P levels in predicting incident CVD events.

Results of meta-analysis showed that the pooled HR (or OR) for per SD increment and Q4 versus Q1 of blood HDL-P level was 0.82 and 0.65, 95% CI ranging from 0.76 to 0.87 and 0.57 to 0.75, respectively. Same as established theories, our data indicates that higher HDL-P level maight be a protective factor for CVD events.

We also analyzed clinical value of different HDL-P subfranctions in predicting CVD events. HRs for per SD increment of large, medium and small HDL-P were 0.90, 0.91 and 0.90, with no significant difference. But the results of Kim’s study [16], small and medium HDL-P were significantly and inversely correlated with carotid intima-media thickening measurement results. The possible reason was that the relation between small and medium HDL-P with cardioprotective paraoxonase1 activity may represent the function of HDL. But HRs for Q4 versus Q1 were 0.91, 0.70 and 0.63, respectively. And 95% CI of HRs for Q4vsQ1 cover 1. One possible explanation was the shortage of studies included. Four [5, 8, 9, 14] studies explored the association between large HDL-P levels and CVD events. Only two [8, 9] researches studied medium and small HDL-P.

HDL particles spectrum changes in the whole HDL metabolism. And different HDL-P subclasses have varied functions. Small HDL-P seems to have a stronger anti-inflammatory effect than large HDL-P [17]. Large HDL-P is reported to have the property of inhibiting platelet activation and stimulating the activation of anti-coagulant proteins [18]. Since the structure and ingredient of HDL particle vary in the process of HDL metabolism, amounts of HDL-C and apolipoprotein A1 differ in different HDL particles. Large HDL-P contains more cholesterol inside. So HDL-C may not reflect the whole function and amount of HDL in blood. HDL-P may be a promising biomarker for CVD events and a new target of therapy.

Heterogeneity existed in pooled HR for per SD increment, but not Q4vsQ1. Sensitivity analysis demonstrated removal of Akinkuolie’s [5] and Parish’s [13] studies made heterogeneity insignificant. No publication bias was observed. With 63,064subjects included, total sample size was large in this meta-analysis study. Our study came to a robust result that blood HDL-P concentration is a promising biomarker reversely related to CVD events, regardless of patients’ basic status.

## Abbreviations

BMI: Body mass index
CHD: Coronary heart disease
CI: Confidence interval
CVD: Cardiovascular disease
EPIC: European Prospective Investigation into Cancer and Nutrition
HDL: High density lipoprotein
HIV: Human immunoddficiency virus
HPS: Heart Protection Study
HR: Hazard ratio
hsCRP: High sensitivity C-Reactive Protein
MDC-CC: Malmö Diet and Cancer Study
MESA: Ulti-ethnic study of atherosclerosis
MI: Myocardial infarction
MRFIT: Multiplle Risk Factor Intervention Trial
NMR: Nuclear magnetic resonance
NOS: NewCastle-Ottawa Quality Assessment Scale
OR: Odds ratio
SD: Standard deviation
TG: Triglyeride
VA-HIT: Veterans Affairs High-Density Lipoprotein Intervantion Trial
WHI-OS: Women’s Health Initiative Observational Study
WHS: Women’s Health Study
